# Draft Genome Sequence of *Paludibacter jiangxiensis* NM7^T^, a Propionate-Producing Fermentative Bacterium

**DOI:** 10.1128/genomeA.00667-17

**Published:** 2017-07-20

**Authors:** Yan-Ling Qiu, Dieter M. Tourlousse, Norihisa Matsuura, Akiko Ohashi, Yuji Sekiguchi

**Affiliations:** a College of Environmental Science and Engineering, Qingdao University, Qingdao, Shandong Province, People's Republic of China; b Biomedical Research Institute, National Institute of Advanced Industrial Science and Technology (AIST), Tsukuba, Ibaraki, Japan; c Faculty of Environmental Design, Kanazawa University, Kanazawa, Ishikawa, Japan

## Abstract

We report here a high-quality draft genome sequence of *Paludibacter jiangxiensis* strain NM7^T^, a mesophilic, anaerobic, propionate-producing fermentative bacterium within the family *Porphyromonadaceae* of the phylum *Bacteroidetes*. The genome comprises 3,664,884 bp in four contigs, has a G+C content of 42.92%, and contains 2,949 protein-coding sequences and 62 RNAs.

## GENOME ANNOUNCEMENT

*Paludibacter jiangxiensis* strain NM7^T^ (JCM 17480^T^, CGMCC 1.5150^T^, KCTC 5844^T^) is a mesophilic, anaerobic, propionate-producing fermentative bacterium (1) and represents one of two currently described species of the genus *Paludibacter* in the family *Porphyromonadaceae*. Strain NM7^T^ shares 91.4% 16S rRNA gene sequence identity with *P. propionicigenes* strain WB4, the type species of the genus *Paludibacter* (2). Both *P. jiangxiensis* strain NM7^T^ and *P. propionicigenes* strain WB4^T^ were isolated from plant residue in anoxic rice paddy field soil and characterized as strictly anaerobic, rod-shaped, and nonmotile bacteria (1, 2). Phenotypic differences exist between both species in terms of their substrate utilization and temperature growth range: *P. jiangxiensis* can utilize sucrose and lactose as sole substrates, whereas *P. propionicigenes* cannot; growth of *P. jiangxiensis* occurs optimally at 37°C, while *P. propionicigenes* grows optimally at 30°C and fails to grow at 37°C (1, 2). Here, we report a high-quality draft genome sequence of *P. jiangxiensis* NM7^T^ to complement the genome sequence of *P. propionicigenes* WB4^T^ reported previously (3).

Pure culture genomic DNA was obtained by phenol-chloroform extraction, and Illumina sequencing libraries were prepared using a Nextera XT DNA library prep kit (400- to 600-bp inserts) and a Nextera mate-pair library preparation kit (1- to 14-kb inserts). The Illumina NextSeq 500 platform with the mid-output kit (300 cycles) was used for sequencing, at coverages of 843× and 105× for the paired-end and mate-pair libraries, respectively. Raw reads were quality filtered using Trimmomatic version 0.32 (4). Filtered paired-end reads were merged with FLASH version 1.2.11 (5), and mate-pair reads were further processed with NextClip version 1.3.1 (6), recovering reads in categories A, B, and C. Assembly was performed using SPAdes version 3.6.0 (7), followed by scaffolding and refinement of the assembly as described previously (8). Annotation of the genome was performed within the Integrated Microbial Genomes (IMG) platform (9).

The final genome assembly of *P. jiangxiensis* NM7^T^ contains four contigs and has an estimated coverage of 948×. The total assembly size is 3,664,884 bp, which is similar to the genome size of *P. propionicigenes* WB4^T^ (3,685,504 bp, NC_014734.1). The genome’s G+C content is 42.92%, which is slightly higher than that of *P. propionicigenes* WB4^T^ (38.86%). Annotation predicted 2,949 protein-coding genes and 62 RNA genes. The majority of the protein-coding genes (74.6%) could be assigned a putative function, and three complete sets of rRNAs were identified. Of note is that while genome annotation indicated that strain NM7^T^ harbors genes encoding lipoproteins required for gliding motility in the phylum *Bacteroidetes* (such as GldC, GldE, GldH), motility was not observed in our previous study (1). We anticipate that the availability of a high-quality draft genome sequence of *P. jiangxiensis* strain NM7^T^, together with genomes of other *Porphyromonadaceae* bacteria, will shed light on the metabolic potential and possible ecological role of members of the family *Porphyromonadaceae* in different ecosystems.

### Accession number(s).

The draft genome sequence of *P. jiangxiensis* NM7^T^ has been deposited at DDBJ/EMBL/GenBank under accession number BDCR00000000 (BioProject PRJDB4691). The version described in this paper is the first version, BDCR01000000.
